# The Impact of Various Cockpit Display Interfaces on Novice Pilots’ Mental Workload and Situational Awareness: A Comparative Study

**DOI:** 10.3390/s24092835

**Published:** 2024-04-29

**Authors:** Huimin Tang, Boon Giin Lee, Dave Towey, Matthew Pike

**Affiliations:** 1 School of Computer Science, University of Nottingham Ningbo China, 199 Taikang East Road, Ningbo 315100, China; huimin.tang@nottingham.edu.cn (H.T.); boon-giin.lee@nottingham.edu.cn (B.G.L.); dave.towey@nottingham.edu.cn (D.T.); 2 Nottingham Ningbo China Beacons of Excellence Research and Innovation Institute, Ningbo 315101, China

**Keywords:** observation study, mental workload, situational awareness, physiological data, instrumentation design

## Abstract

Future airspace is expected to become more congested with additional in-service cargo and commercial flights. Pilots will face additional burdens in such an environment, given the increasing number of factors that they must simultaneously consider while completing their work activities. Therefore, care and attention must be paid to the mental workload (MWL) experienced by operating pilots. If left unaddressed, a state of mental overload could affect the pilot’s ability to complete his or her work activities in a safe and correct manner. This study examines the impact of two different cockpit display interfaces (CDIs), the Steam Gauge panel and the G1000 Glass panel, on novice pilots’ MWL and situational awareness (SA) in a flight simulator-based setting. A combination of objective (EEG and HRV) and subjective (NASA-TLX) assessments is used to assess novice pilots’ cognitive states during this study. Our results indicate that the gauge design of the CDI affects novice pilots’ SA and MWL, with the G1000 Glass panel being more effective in reducing the MWL and improving SA compared with the Steam Gauge panel. The results of this study have implications for the design of future flight deck interfaces and the training of future pilots.

## 1. Introduction

The number of in-service airplanes has increased in the past 20 years, according to data from GAMA (https://gama.aero/wp-content/uploads/2021ShipmentReport-2022-05-16-1.pdf (accessed on 6 July 2022)). It follows that future air traffic will be more congested, increasing the number of factors that could lead to accidents ((https://www.iata.org/en/iatarepository/publications/economic-reports/global-outlook-for-air-transport---december-2023---report/ (accessed on 19 April 2024)). In addition, increased congestion will lead to pilots having to process more information from air traffic controllers, which will increase the number of parallel tasks they need to perform [1]. Mental workload (MWL) is a psychological construct modeled on real activity [2] to describe how much mental effort a given task requires at a given time [3]. The majority of recent aviation accidents have seen high MWL levels [4], highlighting the importance of pilots’ MWL in Flight Operation Safety Assessment (FOSA). Situational awareness (SA) [5] is another important factor in ensuring flight safety [6]. It represents operators’ understanding of a specific situation and can be viewed as a three-step cognitive concept comprised of (1) *perception*, (2) *comprehension* and (3) *projection* [2]. SA-friendly systems improve pilot efficiency and increase flight safety [7].

The airplane’s cockpit display interface (CDI) is one of the most important devices for pilot–airplane interaction [8]. The CDI provides critical airplane state information, such as the heading, altitude and speed. Endsley [5] pointed out that the CDI needs to support pilots in building sufficient SA to maximize flight safety. Existing studies have shown the importance of interface design on operators’ MWL [9]. However, current studies mainly focus on how to design the CDI to better support pilots’ SA [10] or focus on how individual parts of the CDI, such as the navigation system, influence pilots’ MWL [11,12], and rarely is the relationship between a CDI’s support for SA and pilots’ MWL considered.

Two research questions were proposed for this study to study how different subparts of the CDI influence pilots’ SA and how the impact on SA influences their MWL:

RQ1: Do different designs of CDIs that provide the same information have different effects on a pilot’s SA?
RQ2: What impact do different designs of CDIs with the same information have on pilots’ MWL?

This study focuses on a specific combination of several displays on a CDI can provide pilots with spatial awareness to study the proposed RQs. Spatial awareness has been noted as one of the most important components of SA for aviation [13], which can be provided by three orientation variables—pitch, row and heading—and three position variables: altitude, lateral path deviation and flight path location. In this study, the impacts of the selected displays’ layouts on two specific Cessna-172 series CDI types, the Steam Gauge (SG) panel and the G1000 Glass (GG) panel, on novice pilots’ SA and MWL were compared with each other using objective and subjective measures. The GG panel is a digital flight display that integrates all key flight data into a single display, while the SG panel displays information using gyroscopes. The experiment asked the participants to complete a flight task as a primary task and finish a parallel subtask to simulate a real-world flight scenario. The 4D multiple resources model [14] and the resources–performance relationship [15] were adopted to interpret the impact of the CDI design on participants’ MWL and SA. Our study reaffirms the previous finding that the CDI design influences pilots’ SA [8,16,17] and reveals how differently designed CDIs will influence pilots’ MWL. Additionally, this work provides a further demonstration of applying MWL measures in the evaluation of real-world flight displays. Future flight displays can be informed by the results of this study, and CDI designers can understand the impact of different display designs on pilots’ SA and MWL using the methodology employed in this study.

## 2. Related Work

Two aspects will be discussed in this section: (1) situational awareness (SA) and mental workload (MWL) and (2) information processing models.

### 2.1. Situational Awareness and Mental Workload

SA is the assessment of operators’ correctness in comprehending a given situation [2]. It has been modeled as a three-step process: (1) perceiving the elements in an environment during a period (*perception*), (2) comprehending their meaning (*comprehension*) and (3) projecting their following status based on the understanding of the current situation (*projection*) [5]. Existing studies have identified a relationship between the CDI design and pilots’ SA [8,16,17]. During a computer-based flight simulation, Andre et al. [16] presented navigation displays in three display formats and two color-coding types (six trials). They investigated the influence of an inside-out pilot-centered navigation display, an outside-in world-centered navigation display and a 3D outside-in navigation system on novice pilots’ SA. The navigation displays were shown both in monochrome and color. Participants were instructed to accumulate as many points as possible in each of the six trials. The study found that the outside-in navigation display had a more positive effect on pilots’ SA compared with the other display formats, and the use of color in displays improved situational awareness, particularly during emergency situations. Wei et al. [8] evaluated participants’ SA levels for three differently designed GG panels by having them monitor an aircraft’s instrument panel for 12 min, during which a complete flight was simulated (takeoff, climb, cruise, descent and landing). The instrument panel was programmed to freeze at preset intervals during the task, and the participants were queried on their awareness of the aircraft’s current status through a series of questions. They found that the CDI design had a significant impact on pilots’ SA, especially during the *projection* and *comprehension* stages. The SA under Boeing’s CDI was significantly higher than that under Airbus’s at the *projection* stage and significantly lower at the *comprehension* stage. Their findings suggested that although Boeing’s CDI helped participants perceive more information, it was more challenging for them to combine the gathered information for understanding.

MWL can be used in evaluating the performance of the operator and the system [3] to improve the system design. Longo [18] emphasized that excessively high or low MWL would negatively influence human performance. Studies have demonstrated a similar relationship between flight performance and MWL [19,20,21]. Morris and Leung [19] asked different groups of novice pilots to finish different numbers of parallel tasks on a computer-based simulator that simulated a real-world flight task’s different demand levels. The primary task required the participants to keep two crosshairs in the center of the screen using a joystick. The study found a decline in the participants’ primary task performance under a higher MWL.

As reported by Wei et al. [20], the accuracy of manipulating the flight simulator decreased, while the reaction time increased as the pilots’ MWL increased. A limited number of studies have examined the relationship between CDI design and pilots’ MWL, with a focus on the navigation system (primary flight display) on the CDI. For example, Beringer and Ball [12] and Uenking and Hughes [22] investigated the optimal placement of the primary flight display, and Uenking and Hughes [22] and Davis et al. [23] examined ways to design it to minimize MWL. Both Davis et al. [23] and Uenking and Hughes [22] found that the new primary flight displays resulted in a lower MWL compared with conventional round dial instrumentation. Beringer and Ball [12] suggested that the field of view used for the primary flight display should not be less than 40 degrees. However, few studies have examined the effect of the entire CDI design on a pilot’s MWL. Casner [11] compared the impact of the CDI with round dial and electronic instruments on pilots’ MWL, but the small sample size precluded clear conclusions.

Studies have highlighted the interaction between SA and MWL and its effect on pilots’ performance [8,24]. Lin and Lu [24] designed a simulated helicopter rescue task. The task difficulty was induced by different emergencies, such as some suddenly broken instruments or random alarm lights on the panel. They found that low SA led to a high MWL, and decreased pilots’ performance, especially for unskilled pilots.

### 2.2. Information Processing Models

The 4D multiple resources model (MRM) proposed by Wickens [25] describes three stages of information processing: *perception*, *cognition* and *responding*. Different dimensions, such as the *visual* and *auditory* dimensions, might be involved in information processing, depending on the task demands, and consume the corresponding types of cognitive resources. This model is especially suitable for predicting probable inter-task interference during multitasking [25], meaning designers can predict such interference in advance [26] and identify resources demand conflicts [27]. Existing studies have also established that this model can accurately represent changes in MWL during multitasking [28]. When the tasks require resources from the same dimension, such as several tasks requiring *audio* resources to handle different sounds, the operator’s MWL will be greater relative to task demands from differing resource dimensions. Resource competition happens frequently during piloting [14]. For example, the competition for *visual* resources is intense due to the need to process information from a large number of instruments [29] or the requirements when simultaneously perceiving information from both the instruments and the outside environment [30]. The MRM has been widely used in exploring the relationship between the pilots’ workload and their performance [31]. Thorpe et al. [31] used the MRM to explain their findings that the negative effect of two parallel tasks requiring different resources (visual aviation task and auditory secondary task) was less than that of two parallel tasks requiring the same resources (visual aviation task and visual secondary task) on pilots’ flight performance.

**Figure 1:**
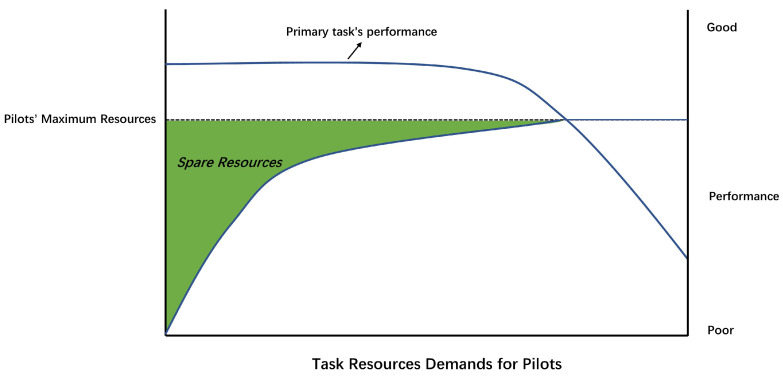
Resources–performance relationship, adapted from Sharples and Megaw [15] (p. 520).

The resources–performance relationship adapted by Sharples and Megaw [15] (p. 520) showed that the human capacity for processing information is limited (Figure 1). When the spare capacity approaches zero, primary task performance will decline. Mental overload occurs if the required resources exceed the available resources ([15], p. 543). Combined with the MRM [25], when several tasks (including the primary task) require resources from the same dimension, the remaining capacity in this dimension declines, increasing the possibility of primary task performance degradation. Therefore, resource competition in one dimension could induce both a higher MWL and worse performance. This has been shown in existing pilot-related studies [8,32].

## 3. Methods

### 3.1. Hypotheses

**Figure 2:**
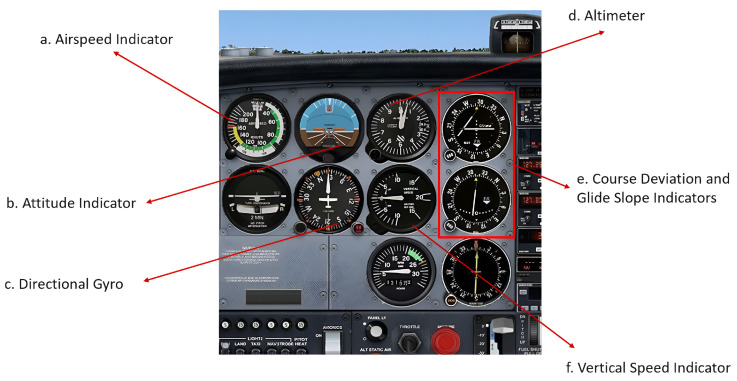
The Cessna 172 Steam Gauge (SG) panel.

**Figure 3:**
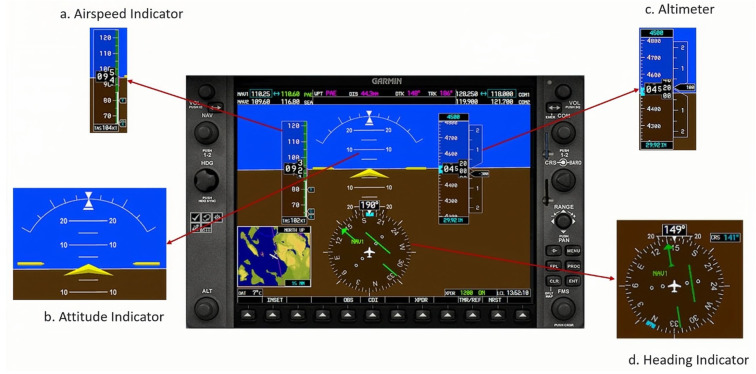
The Cessna G1000 Glass (GG) panel.

Pilots will consume fewer cognitive resources on building up spatial awareness if the displays related to these variables are appropriately constructed. Wickens [13] stated that maintaining SA can be viewed as a cognitive task that requires cognitive resources to process selective information and subsequently may impact pilots’ MWL. The Cessna 172 SG panel (Figure 2) and the Cessna 172s GG panel (Figure 3) were chosen for this study because of their widespread use and distinctive differences in spatial awareness-related instrumentation design, including the airspeed indicator, altimeter, attitude indicator and heading indicator ((a–d) in Figure 2 and Figure 3).

The following hypotheses were developed based on the research questions:

- **HPSA** (Operator’s SA)
  Under the same task difficulty, there will be a significant difference in SA between two different CDIs.
- **HPMWL** (Operator’s MWL)
  For the same task, the operator’s MWL will be significantly lower with a CDI that has a higher SA level than with a CDI that has a lower SA level.

**HPSA** is proposed to address RQ1 and is based on the findings of Wei et al. [8], who identified a relationship between different CDI designs and their impact upon pilots’ SA (discussed in Section 2.1). In addition, Endsley [33] pointed out that the system interfaces that were designed based on SA-oriented interface design principles [33] have been shown to better support operators’ SA, suggesting the interface design’s impact on the operator’s SA. Compared with the digital display, the pointer-type ones used on the SG panel have a higher possibility of misreading, especially when the operator is asked to make quantitative readings from the display [34]. Such displays have a higher possibility of depleting the operator’s resources for maintaining sufficient SA.

**HPMWL** was drawn from the resources–performance relationship ([15], p. 520) and MRM [25] for exploring RQ2. Facing limited resources, operators will theoretically have more spare capacity for other parallel cognitive tasks and have less intense competition in resources when SA can be maintained from the CDI using fewer resources, especially when the parallel tasks require resources from the same dimension as maintaining SA. This decreases the possibility of a high MWL and mental overload. A lower MWL may result from the CDI that better supports the pilot’s SA.

### 3.2. Task

#### 3.2.1. Primary Task

**Figure 4:**
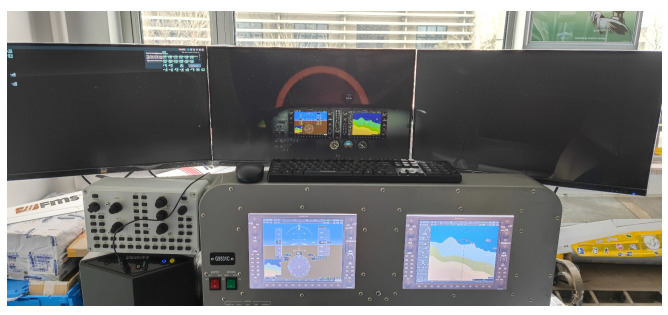
Flight simulator and environment for primary task.

A flight simulator running *Microsoft Flight Simulator 2020* was provided by the School of Aerospace at the University of Nottingham Ningbo China (UNNC) and was used to conduct the primary task. Participants were required to pilot the airplane, maintaining a heading of 90° E at an altitude of 2500 feet (0.76 km) for 4 minutes. The task was completed at nighttime in the simulation environment, meaning that there were little-to-no visual landmark cues for participants to utilize. Instead, the participants had to rely solely on the readings from the CDI (Figure 4) to complete the task.

There were several considerations in the primary task design to reduce the influence of factors unrelated to the CDI. The task tried to reduce extra operations from the task that might distract participants, such as communicating with the traffic controller. Because this study focused on the instruments that provide spatial awareness of the CDI, the tasks were designed such that the participants were required to regularly consult the CDI.

#### 3.2.2. Secondary Task

The N-back task [35] was the secondary task, which required the participants to speak out the number that previously appeared n items ago in the task. At the **perception** stage, referring to the MRM [25], both the primary and N-back tasks needed to use **visual** resources, making the competition in the *visual* dimension become intense and resulting in a significant increase in the participants’ MWL. Based on the resources–performance relationship ([15], p. 520), the use of *visual* resources by the N-back task resulted in a decrease or even a complete consumption of spare resources for the primary task, leading to a degradation in the primary task’s performance. Based on the resources–performance relationship ([15], p. 520), the utilization of *visual* resources by the N-back task led to a partial or total depletion of spare resources for the primary task, resulting in a decline in primary task performance. At the *cognition* stage, the competition for *verbal* resources between the N-back task and the primary task became more intense as the N-back task’s difficulty increased. This implies that as the difficulty of the N-back task increases, primary task performance may degrade, and mental workload (MWL) may increase. Hence, we expected the following:

- As the difficulty of the N-back task increased, the performance of the primary task degraded.
- As the difficulty of the N-back task increased, the MWL of the participants rose.

Participants were required to complete three rounds of tasks with each CDI:

- Single flight;
- Flight + one-back task;
- Flight + two-back task.

A “baseline” task of sitting still for one minute was conducted at the beginning of the study to record the participants’ physiological data that could reflect their MWL (discussed further below) during a resting state. The order of the six tasks for each participant was arranged using Latin square rotation to minimize the effects of the task order on the results [36] (see Table 1). The number of participants was larger than six. Thus, the task order started from the beginning of the table again after one round.

### 3.3. Participants

Ten participants (5 male and 5 female) with an average age of 21 years were recruited to take part in the experiment. The participants were all from UNNC. All participants had normal or corrected vision and reported no history of head trauma or brain damage. All the participants had less than 20 h of flight experience on flight simulators but no experience with real-world piloting to ensure there would not be confounding variables caused by different experience levels or different educational backgrounds. The study was approved by the faculty’s ethics committee, and all participants provided informed consent prior to beginning the study.

### 3.4. Data Collection

*PsychoPy* (https://www.psychopy.org/ (accessed on 21 April 2021)) was used to present the N-back task and record associated performance data as well as to stream sensor data using the lab streaming layer (LSL) protocol (https://github.com/sccn/labstreaminglayer (accessed on 21 April 2021)). *LabRecorder* (https://github.com/labstreaminglayer/App-LabRecorder (accessed on 21 April 2021)) was used to receive the streams transferred via the LSL and record them together into an XDF file with time synchronization between them onto the researcher’s laptop as the study was being completed.

**Figure 5:**
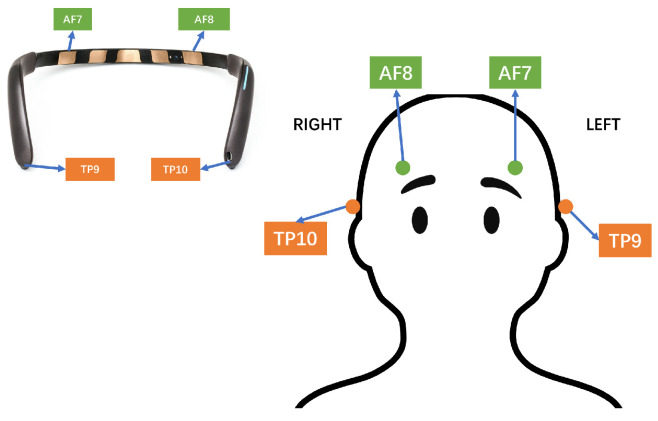
*Muse2* sensor locations using the 10-20 EEG placement system.

Changes in MWL have been shown to be reflected in changes in electroencephalography (EEG) components. Among them, the θ band (4∼7 Hz) and α band (8∼12 Hz) [37] have been successfully used in many pilots’ MWL-related experiments (e.g., [38]). Several studies have found that the α band decreased in the frontal and central regions of the cerebral cortex [39] and parietal regions [40], whereas the θ band increased as the MWL increased, especially in the frontal regions [41]. *Muse2* (https://choosemuse.com/muse-2/ (accessed on 21 April 2021)), a noninvasive EEG headband with four sensors on the forehead and ears, and a sampling rate of 256 Hz were used in this study. EEG measures electrical activity originating from different brain regions by placing sensors over the scalp [37]. The headband sits on the subject’s forehead (locations AF7 and AF8) and rests on the ears (TP9 and TP10), as shown in Figure 5, using the international 10-20 EEG placement system [42]. Several studies have raised concerns regarding the accuracy and correctness of *Muse2*’s readings (e.g., [43]), but the device remains popular because it is highly portable, minimally invasive, easy to use and low in cost, especially when compared with clinical EEG systems [43]. *Muse2* has been proven to be sensitive in monitoring users’ brain activities [44]. *BlueMuse* (https://github.com/sccn/xdf/releases/tag/v1.12 (accessed on 21 April 2021)) was used to collect data from *Muse2* and to interface with the LSL.

Heart rate variability (HRV) is another common physiological measure and has a known relationship with MWL [45]. The results of spectral analysis on HRV show that the low-frequency band (LF, 0.04∼0.15 Hz) represents cardiac sympathetic nervous activities, which are more active under heavy workload and stress, while the high-frequency band (HF, 0.15∼0.40 Hz) relates to the cardiac vagal nervous activities, which are in charge of cardiac activities in normal cases [37]. The LF/HF ratio, representing the balance of the sympathetic tone and vagal tone, can be used as a reliable index to reflect the changes in MWL [46]. According to Murata [47], the LF/HF ratio increases as the MWL increases. A *Polar H10* (https://www.polar.com/uk-en/sensors/h10-heart-rate-sensor (accessed on 21 April 2021)), a sports chest strap, was used to capture the participants’ HRV during the study. The H10 is an ECG device that has been validated in previous studies measuring HRV under naturalistic task conditions [48]. *BLEPolarDirect* (https://github.com/roberttwomey/nosc-toolkit (accessed on 21 April 2021)) was used to pass the data returned (via Bluetooth) from the *Polar H10* to *PsychoPy* via the LSL.

### 3.5. Procedure

**Figure 6:**
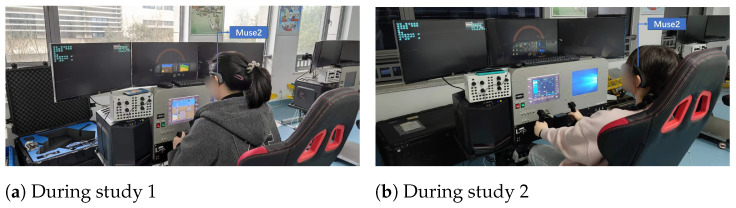
During study.

Before the start of the study, the participants were given 20 minutes to familiarize themselves with the flight simulator. At the beginning of the study, the participants were asked to sit still and relax for one minute while their physiological data were recorded using *Muse2* and the *Polar H10*. The baseline data were used to ensure the validity of the subsequent data collected and to eliminate noise in the EEG data. At the beginning of each task in the study, the airplane in the flight simulator was set to a starting altitude of 2500 feet (0.76 km) and an airspeed of 163 knots, heading in the direction of 90 degrees east. This airspeed is typical for a Cessna 172 series airplane [49]. The participants were expected to maintain the same altitude and direction during the experiment (Figure 6). Maintaining the airspeed requires pilots to consider various factors such as outside weather conditions, wind speed, air friction and the airplane’s engine power. Although the weather and wind speed were constant in this study, changes in altitude have an impact on air drag and engine power and vice versa, resulting in changes in airspeed. As the participants in the study were novice pilots and may not have had the ability to maintain both the airspeed and altitude, they were not required to control the airspeed during the experiment.

**Figure 7:**
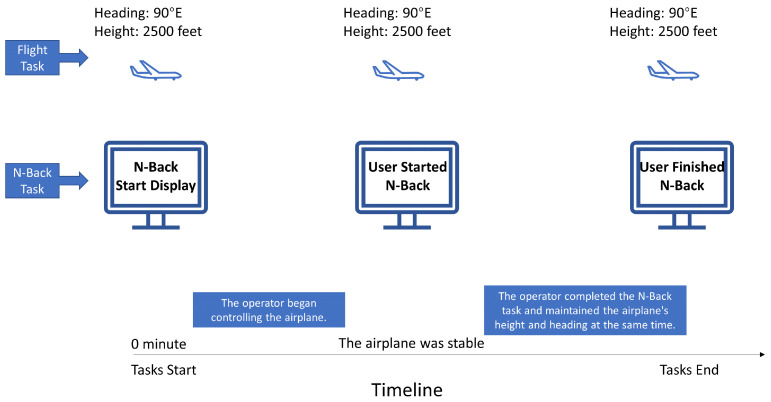
Procedure.

The participants were required to perform an N-back task concurrently with a primary flight task. The N-back task was initiated at the same time as the flight task, and the numbers for the task were displayed on a separate screen as the participant started to operate the flight simulator. There was no set time for when the participants should start the N-back task, and they were able to begin it whenever they deemed their airplane to be stable. The participants were asked to verbally provide the answer to the N-back task. When they completed the N-back task, the flight task ended (Figure 7). The N-back answers, EEG data and HRV data were recorded using *PsychoPy*, while the flight performance data were recorded by the flight simulator.

After each task, the participants completed the NASA Task Load Index (NASA-TLX) assessment to evaluate their experience during the task. The NASA-TLX is a commonly used subjective MWL measure [50]. It has been shown to be quite reliable [51] and has been used extensively in aviation contexts [1,32]. The NASA-TLX predicts MWL by asking the participants to subjectively evaluate six linear scales: mental demand, physical demand, temporal demand, performance, effort and frustration.

After completing the assessment, the participants took one minute of relaxation time before beginning the next task. This process was repeated for all subsequent tasks.

## 4. Results

In this study, all of the dependent variables (flight performance, NASA-TLX score, HRV and EEG) were determined to be non-parametric data based on the normal distribution of their values. All data were determined to be distribution-free using a Shapiro–Wilk test (*p* < 0.05). All dependent variables were either ordinal (NASA-TLX score) or continuous (flight performance, HRV and EEG) data. Therefore, the Wilcoxon test was used to examine the impact of the type of CDI (two levels) on the dependent variables, while the Friedman test was applied to address the influence of the difficulty of the N-back task (three levels) on the dependent variables [52]. Median rather than mean values were used in the data analysis because of the relatively small sample size and to reduce outlier effects [53].

### 4.1. Flight Performance

The participants were required to maintain the airplane’s altitude and heading. Consequently, the flight performance criteria were based on the time the operators kept the airplane at different altitudes and headings during the task (Table 2).

After applying the scoring criteria, a final score for each participant was calculated as follows:

(1) $$\begin{array}{cc} \; & scoreList=[5,3,2,0] \end{array}$$

(2) $$\begin{array}{cc} \; & performanceList=[Perfect,Good,Normal,Poor] \end{array}$$

(3) $$\begin{array}{cc} \; & rating=\frac{time\;of\;keeping\;at\;this\;performance\;level}{total\;flight\;time} \end{array}$$

(4) $$\begin{array}{cc} \; & finalScore=\sum_{i=1}^{4}rating_{performanceList[i]}\times scoreList\left[i\right] \end{array}$$

The flight task required the operator to have sufficient SA about the airplane because its altitude and heading would continually change based on real-time spatial awareness. For example, the operators needed to understand that the current data on the CDI indicated a “downtrend”, and they needed to make the right decision based on the information on the CDI to keep the altitude from changing. Therefore, flight performance acted as the mapping of the SA level in this study.

**Figure 8:**
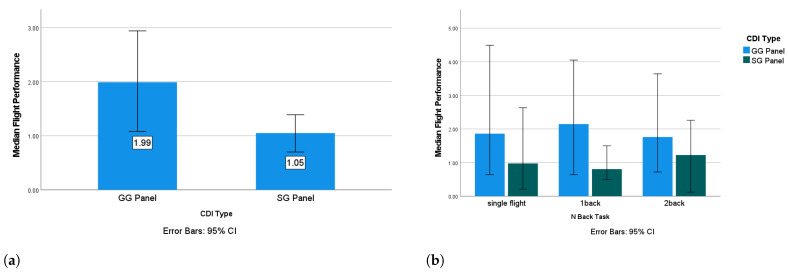
Median flight score under different conditions. (**a**) Median flight scores for different CDIs; (**b**) Median flight scores grouped by CDI and N-back conditions.

The flight performance with the GG panel was significantly better than on that with the SG panel (Z=−3.816,p<0.001), which supports the hypothesis that the CDI type would have an impact on flight performance (**HPSA**) and was consistent with previous findings [8,16,17]. The overall better flight performance with the GG panel (Figure 8a) suggests that the GG panel is the more SA-friendly CDI compared with the SG panel. No significant impact from the difficulty of the N-back task on flight performance was detected. To further examine the impact of the CDI type on flight performance under different N-back conditions, we conducted follow-up Wilcoxon tests on flight performance grouped by CDI type under each of the three N-back conditions. The results revealed significantly better flight performance with the GG panel under the single-flight (Z=−2.395,p=0.017) and one-back (Z=−2.497,p=0.013) conditions, but there was no significant difference between the CDI types under the two-back condition. This suggests that when the multi-task condition does not exceed the operator’s limited cognitive resources, the interface design has a significant influence on their SA level. However, when participants experience an extremely high MWL or mental overload (such as in the two-back condition in this study), they may make task management errors [54], causing the flight task to become their secondary task and resulting in a lack of cognitive resources. This may explain the non-significant difference in SA level between the two CDI types under the two-back condition. Despite this, the median scores still showed that the SA level was higher when using the GG panel compared with the SG panel under the two-back condition (Figure 8b), suggesting that the GG panel is the more SA-friendly CDI.

### 4.2. MWL Measures

#### 4.2.1. HRV Data

**Figure 9:**
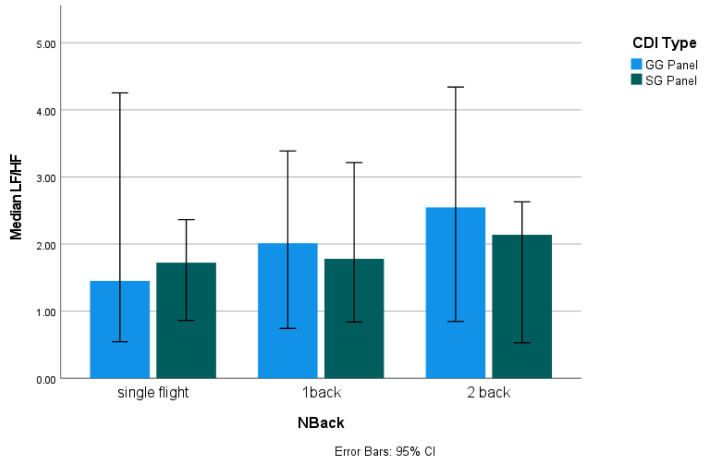
Median LF/HF ratio under different conditions.

The *hrvanalysis* library in Python [55] was used to calculate the LF/HF ratio. No significant differences in the LF/HF ratio were found between the different CDI types or N-back conditions. There was also no clear trend in the HRV data between the different conditions (Figure 9). This may be because the flight task in this study was designed to be a “cruise” stage, which is typically associated with relatively low cognitive demands. According to Wilson [21], HRV data tend to have more noticeable changes during the takeoff and final landing stages of a flight, while the HRV data during other stages of the flight may only exhibit slight changes. HRV may not be sensitive enough to identify differences in MWL under different task demands if the demand changes are not large enough [56].

#### 4.2.2. EEG Data

The EEG data were first channel re-located based on the international 10–20 system using *EEGLab* (https://github.com/sccn/eeglab (accessed on 21 April 2021)) to ensure the channel names in the data file had the correct correspondence with the locations on the scalp. Then, the signals were re-referenced using EEGLab to ensure the signals in each channel were not impacted by the different distances between the sensors and the reference sensor. The mean mastoids (MMs) of TP9 and TP10 were used as the re-referencing point, which is a widely used re-referencing method [57]. Then, after filtering the EEG data using a 1–30 Hz filter, a wavelet transform [58] was applied to extract the α band and θ band required for this study from the original EEG signal, which is a method for decomposing the EEG signal into its frequency components through dilatations, contractions and shifts [59]. Additionally, the wavelet transform is suitable for the spectral analysis of each decomposed frequency band, making it easier to calculate the relative energy of the bands.

##### The θ Band

**Figure 10:**
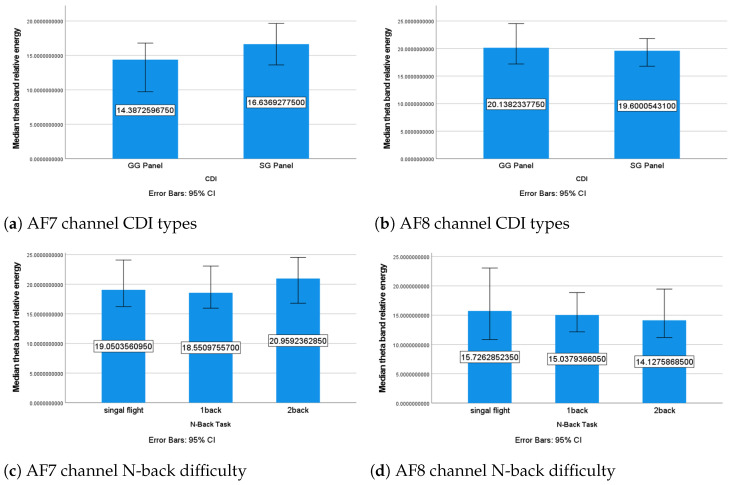
Median θ band’s relative energy under different conditions.

The statistical analysis revealed that the CDI type and N-back task did not have a significant effect on the relative energy of the θ band in either the AF7 and AF8 channels. The overall relative energy of the θ band was lower under the GG panel and highest under the two-back condition in the AF7 channel (Figure 10a,c). Previous research has shown that when individuals engage in mental activities that require higher cognitive resources, the θ power tends to increase more over the frontal cortex area of the brain [60], which is the area that the AF7 and AF8 electrodes of *Muse2* correspond to. Therefore, the lower relative energy of the θ band in the AF7 channel when operating the GG panel observed in this study may indicate that the operators had a lower MWL under this condition. This finding supports the conclusion from the flight performance analysis that the GG panel is the more SA-friendly CDI in this study, even though it does not fully support the hypothesis that the MWL would be lower under the GG panel (**HPMWL**). The higher relative energy of the θ band in the AF7 channel under the two-back condition suggests that the two-back task induced a higher MWL than the single-flight and one-back conditions. This is consistent with the conclusion from the flight performance analysis that the non-significant difference between the CDI types under the two-back condition may have been caused by the participants experiencing extremely high MWLs or mental overload. However, the findings in the AF8 channel showed a trend that was opposite to that in the AF7 channel (Figure 10b,d). The relative energy of the θ band was higher for the GG panel, and the single flight induced the highest MWL among the three N-back conditions.

##### The α Band

**Figure 11:**
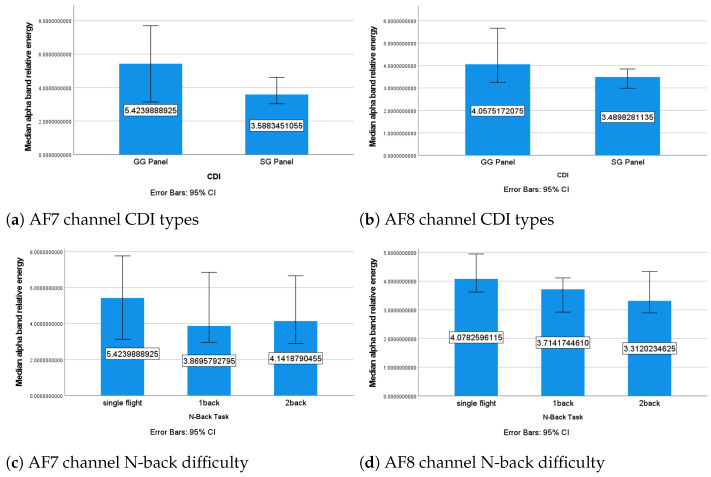
Median α band’s relative energy under different conditions.

When using the GG panel, the relative energy of the α band was significantly higher in the AF8 channel (Z=−2.211,p=0.027) and marginally significantly higher in the AF7 channel (Z=−1.8,p=0.072) compared with the SG panel (Figure 11a,b). When the Wilcoxon test was applied to the relative energy of the α band in the AF8 channel grouped by CDI type under each of the three N-back conditions, a significantly higher α band relative energy for the GG panel was found under the single-flight condition (Z=−2.293,p=0.022), and a marginally significantly higher α band relative energy for the GG panel was found under both the one-back (Z=−2.211,p=0.062) and two-back conditions (Z=−1.886,p=0.059).

The relative energy of the α band showed a significant difference between the different N-back conditions in both the AF7 ($\chi^{2}=6.4,p=0.041$) and AF8 ($\chi^{2}=6.3,p=0.043$) channels. After applying a Bonferroni correction [61], the α band’s relative energy under the single-flight condition was significantly higher than under the one-back condition in the AF7 channel (p=0.034) and significantly higher than under the two-back condition in the AF8 channel (p=0.018) (Figure 11c,d). This suggests that the N-back tasks increased the MWL of the participants compared with the single-flight condition.

The results of this study suggest that the GG panel may be designed to be more spatial awareness-friendly, inducing a lower MWL compared with the SG panel under the same conditions. This was inferred from the decrease in the overall relative energy of the α band in both the AF7 and AF8 channels while using the SG panel, as the α waves’ increment has been shown to reflect a relaxed mental state [62]. The N-back tasks also increased the MWL of the participants compared with the single-flight condition, as indicated by the significantly increased relative energy of the α band in the AF7 and AF8 channels. It is worth noting that there were some marginally significant results (p<0.075), and increasing the sample size may further strengthen these results [63].

##### The θ/α Ratio

**Figure 12:**
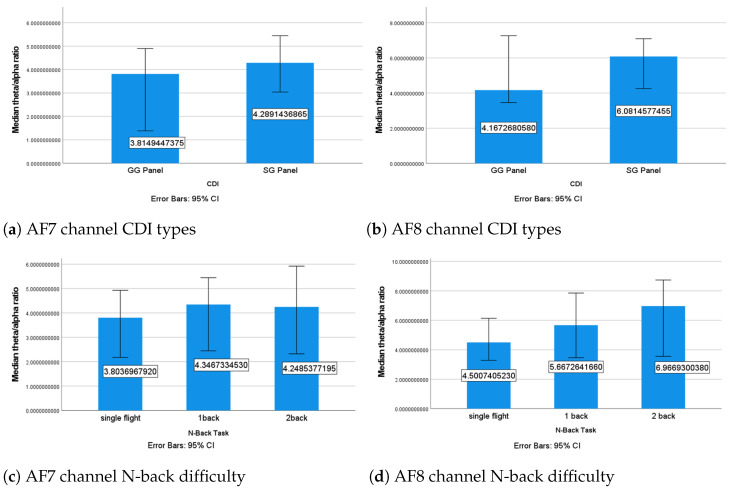
Median θ/α ratio under different conditions.

No significant impact from the CDI types on the θ/α ratio was identified in either channel. The overall θ/α ratio under the SG panel was higher in both channels (Figure 12a,b). Since the increase in MWL can be reflected by the increment of the θ/α ratio [64], we interpreted that the participants’ MWL under the SG panel was descriptively higher than that under the GG panel, which could support the trend in **HPMWL**.

There was no significant difference in the θ/α ratios between the different CDI types in either the AF7 or AF8 channels. The overall θ/α ratio was higher under the SG panel in both channels (Figure 12a,b). Since an increase in MWL can be reflected by an increase in the θ/α ratio [64], it can be concluded that the participants’ MWL was descriptively higher while using the SG panel compared with the GG panel. This finding supports **HPMWL**.

There was no significant difference in the θ/α ratio between the different N-back conditions in the AF7 channel (Figure 12c), but a significant difference was found in the AF8 channel ($\chi^{2}=9.1,p=0.011$). After applying a Bonferroni correction [61], a significant difference was found between the single-flight and one-back conditions (p=0.034) and between the single-flight and two-back conditions (p=0.022) in the AF8 channel. The θ/α ratio under the single-flight condition was significantly lower than the one-back and two-back conditions (Figure 12d). This suggests that the N-back tasks increased the MWL of the participants during the flight task, which is consistent with the findings obtained from the analysis of the α band’s relative energy.

#### 4.2.3. NASA-TLX Score

**Figure 13:**
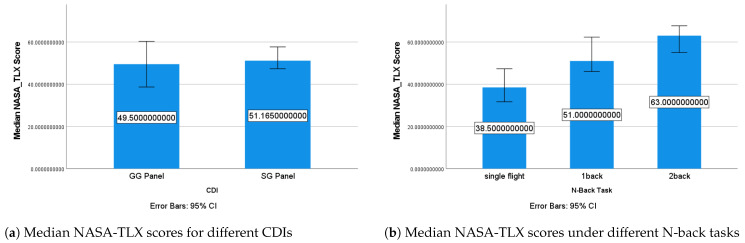
Median NASA-TLX scores under different conditions.

The overall NASA-TLX score was used for analysis. It is worth noting that while there was no significant difference in NASA-TLX scores across the CDI types, there was a slightly higher median score when the participants used the SG panel. This suggests that the SG panel may have caused a slightly higher MWL compared with the GG panel, although the difference was not statistically significant. A significant impact of the N-back condition’s difficulty on the NASA-TLX score was found ($\chi^{2}=29.1,p<0.001$). After applying a Bonferroni correction [61], the NASA-TLX score under the two-back condition was significantly higher than that under the single-flight (p<0.001) and one-back conditions (p<0.001). This suggests that the N-back tasks successfully induced different levels of pressure on the operators, with the overall NASA-TLX score increasing with the difficulty of the N-back task (Figure 13b). This is consistent with the findings in the α band’s relative energy and the θ/α ratio, which also showed an increase in MWL with increasing N-back condition’s difficulty.

### 4.3. Limitations

The participants in this study were novice pilots, which could lead to easier mental overload than for experienced pilots under similar circumstances. To minimize the possibility of mental overload in situations requiring relatively low cognitive demands due to the participants’ unskilled operations, we designed the tasks as cruise tasks and N-back tasks, which were easier to complete compared with other flight stages’ tasks. The impact of the same CDI on pilots might also be different depending on their familiarity level with the cockpit. In future work, the participants can be pilots with various experience levels, the primary task can be a more cognitive resource-dependent one, such as takeoff, and the secondary tasks can be real parallel tasks, such as “communicating with an air traffic controller”. This would be more similar to today’s aviation and improve the study’s universality.

The physiological evaluation tools for MWL could be more comprehensive in future studies. As reflected in the current results, HRV is not sensitive enough to detect changes in MWL when there are no significant changes. The eye-tracking technique can be considered a future evaluation tool. The pupil size change has been highlighted by previous studies as being sensitive enough for MWL measurement [65,66]. The participants’ focused areas can be revealed by the areas of interest captured by the eye tracker [67,68], making it suitable for investigating the specific parts of the CDI that have a noticeable impact on participants’ SA and MWL.

## 5. Conclusions

In this study, we compared the effect of using the G1000 Glass (GG) and Steam Gauge (SG) panels in the Cessna 172 series as cockpit display interfaces (CDIs). The results showed that the GG panel was more situational awareness (SA)-friendly, as the participants had lower mental workloads (MWLs) when using it. This emphasizes the importance of designing SA-friendly interfaces in the aviation industry. However, it is worth noting that MWL can also be affected by other parallel flight tasks involving a CDI.

This study also found that the N-back tasks used in the experiment were successful in inducing different levels of MWL in the participants. These findings have implications for the design of CDIs and the management of MWL in the aviation industry and contribute to all the areas that involve interface design and MWL-SA evaluation.

We believe that the results of this study provide a clear direction for future work, which can be replicated on a larger scale with more diverse groups to improve the representativeness of the study. Our next step is to investigate how the layout design affects MWL under different flight tasks such as takeoff and landing. Additionally, this study successfully demonstrated that measuring SA and MWL simultaneously is viable, providing a direction for future interface design and improvement by using these two aspects as evaluation tools together.

## Figures and Tables

**Table 1:** Experiment order.

	Order of the Experiment					
**Participant**	**1**	**2**	**3**	**4**	**5**	**6**
1	single-flight digital	1 back digital	2 back analogue	2 back digital	1 back analogue	single-flight analogue
2	1 back digital	2 back digital	single-flight digital	single-flight analogue	2 back analogue	1 back analogue
3	2 back digital	single-flight analogue	1 back digital	1 back analogue	single-flight digital	2 back analogue
4	single-flight analogue	1 back analogue	2 back digital	2 back analogue	1 back digital	single-flight digital
5	1 back analogue	2 back analogue	single-flight analogue	single-flight digital	2 back digital	1 back digital
6	2 back analogue	single-flight digital	1 back analogue	1 back digital	single-flight analogue	2 back digital

**Table 2:** Flight performance criteria.

	Heading (°)	Altitude (Feet)
Perfect (5 points)	90 ± 10	2500 ± 100
Good (3 points)	65–80, 100–115	2250–2400, 2600–2750
Normal (2 points)	50–65, 115–130	2100–2250, 2750–2900
Poor (0 point)	Others	Others

## Data Availability

The raw data supporting the conclusions of this article will be made available by the authors on request.
